# Fabrication of Hierarchical ZnO@NiO Core–Shell Heterostructures for Improved Photocatalytic Performance

**DOI:** 10.1186/s11671-018-2676-1

**Published:** 2018-08-30

**Authors:** Meng Ding, Hongcen Yang, Tian Yan, Chenggang Wang, Xiaolong Deng, Shouwei Zhang, Jinzhao Huang, Minghui Shao, Xijin Xu

**Affiliations:** School of Physics and Technology, University of Jinan, 336 Nanxinzhuang West Road, Jinan, 250022 People’s Republic of China

**Keywords:** ZnO@NiO, Electrochemical deposition, Photocatalytic, Photocurrent response

## Abstract

ZnO@NiO core–shell heterostructures with high photocatalytic efficiency and reusability were prepared via electrochemical deposition on carbon fiber cloth substrates. Their photocatalytic properties were investigated by measuring the degradation of rhodamine B and methyl orange (MO) under ultraviolet light irradiation. The photodegradation efficiency of the ZnO@NiO heterostructures toward both dyes was better than those of the pure ZnO nanorods and NiO nanosheets. The higher performance could be attributed to the formation of p–n heterojunction between ZnO and NiO. Especially, the ZnO@NiO heterostructure formed upon deposition of NiO for 10 min degraded 95% of MO under ultraviolet light irradiation for 180 min. The high photodegradation efficiency of the ZnO@NiO heterostructures was also attributed to the high separation efficiency of photogenerated carriers, as confirmed by the higher photocurrent of the ZnO@NiO heterostructures (eightfold) when compared with that of the pure ZnO nanorods. Moreover, the high photodegradation efficiency of the ZnO@NiO heterostructures was maintained over three successive degradation experiments and decreased to 90% after the third cycle.

**Electronic supplementary material:**

The online version of this article (10.1186/s11671-018-2676-1) contains supplementary material, which is available to authorized users.

## Background

Over the past few decades, semiconductor photocatalysis, as a type of “green technology,” has attracted much attention owing to its potential applications in environmental protection and energy production [1–3]. Typical semiconductors that have been investigated are TiO_2_ [4], ZnO [5–7], Cu_2_O [8, 9], CdS [10, 11], and C_3_N_4_ [12]. Among them, ZnO has been the most systemically investigated owing to its high electron mobility, diverse morphologies, ease of preparation, low cost, and non-toxic nature [13, 14]. ZnO, with direct wide bandgap (3.37 eV), usually exhibits n-type conductivity due to the native defects, including zinc interstitials and oxygen vacancies. However, ZnO as a photocatalyst has several limitations: (1) its large bandgap favors the use of ultraviolet light mostly for photodegradation to occur; (2) fast internal recombination of photogenerated electron–hole pairs results in poor photodegradation efficiency [15, 16]; (3) the occurrence of severe photocorrosion during the photocatalytic process hinders effective degradation of organic pollutants. Therefore, the development of high-performance photocatalysis based on ZnO remains a challenge.

Many research groups have looked into improving the separation efficiency of photogenerated carriers and extending the spectral response range via, for example, doping [17], loading noble metals [5, 18–20], and combining with other semiconductors [21–31]. As a potential candidate, nickel oxide, p-type semiconductor material (Eg = 3.5 eV) with a rock salt or cubic structure, has attracted much interest owing to its electronic structure, high hole mobility, and low lattice mismatch with ZnO. Thus, it can be used to fabricate p–n heterojunction with ZnO. Moreover, ZnO@NiO heterojunction can form a type II band structure. The conduction band (CB) of ZnO is located between the valence band (VB) and the CB band of NiO; such a configuration can hinder the recombination of photogenerated electron–hole pairs, potentially leading to improved photocatalytic efficiency. Zhang et al. [32] reported the synthesis of p-type NiO/n-type ZnO heterojunction nanofibers using sol–gel process and electrospinning technology and their use as photocatalysts. The latter exhibited higher catalytic activities than pure NiO and ZnO nanofibers. Luo et al. [33] reported that ZnO nanoneedles directly grown from a porous Ni foam or NiO surface displayed a 2.5-fold higher photocatalytic performance than pure ZnO. Lei et al. fabricated hierarchical porous ZnO/NiO hollow microspheres, which have superior adsorption capacity for Congo [34]. Despite the improved photocatalytic efficiencies reported, the use of current ZnO@NiO heterostructures as photocatalysts still suffer from drawbacks such as complex synthesis process, difficulty in photocatalyst separation from the reaction medium, and subsequent reuse of the photocatalysts. Particularly, the separation of the photocatalysts from the solution after reaction is a challenge in practical photocatalytic processes.

In the present paper, a carbon fiber cloth is chosen as a substrate to synthesize ZnO@NiO heterostructures by electrochemical deposition. Such a configuration allows easy separation of the photocatalyst from the solution and recycling of the photocatalyst. The photocurrent response performance of the hierarchical ZnO@NiO core–shell heterostructures is also discussed.

## Methods

### Materials Preparation

ZnO nanorods were grown on a carbon fiber cloth via an electrochemical deposition method. Prior to use, the carbon fiber cloth was cleaned by sequential sonication in acetone, ethanol, and deionized water. A mixed aqueous solution (electrolyte) of 5 mM zinc nitrate hexahydrate (Zn(NO_3_)_2_·6H_2_O) and 5 mM hexamethylenetetramine, carbon fiber cloth, a 2 cm × 2 cm platinum plate, and Ag/AgCl in a saturated KCl solution were used as working, counter, and reference electrodes, respectively. The electrolytic cell was placed in a water bath to maintain a constant temperature of 90 °C. The reaction was carried out for 30 min at a constant potential of − 0.9 V versus the reference electrode. After the reaction, the samples were washed several times with deionized water and dried in an oven at 60 °C for 24 h.

NiO nanosheets layer was deposited on a carbon fiber cloth via electrochemical deposition, and 0.01 mol nickel nitrate hexahydrate (Ni(NO_3_)_2_·6H_2_O) was dissolved in 500 mL of deionized water. The reaction was carried out at a constant potential of − 1 V versus the reference electrode for 10 min. After the reaction, the samples were taken out of the solution and washed several times with deionized water, followed by annealing at 400 °C in an oven for 2 h in air.

**Fig. 1:**
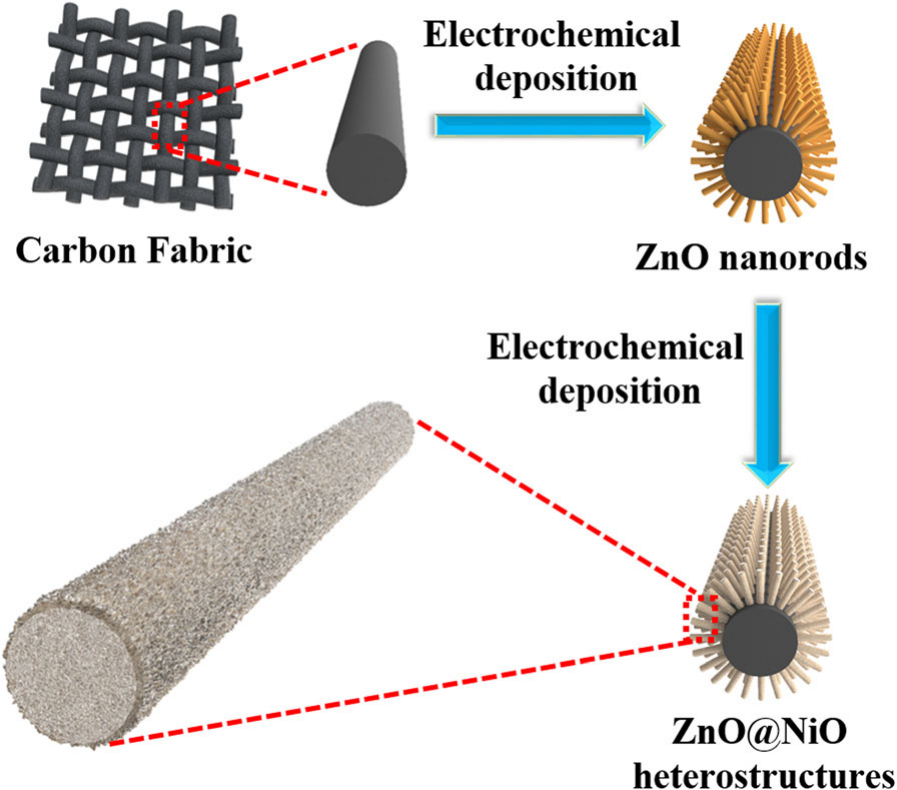
Fabrication process of hierarchically ZnO@NiO heterostructures

To prepare the ZnO@NiO heterostructures, a NiO layer was deposited on the ZnO nanorods via electrochemical deposition. The deposition time was varied from 5 to 10 and 15 min. The resulting samples are respectively denoted as ZN1, ZN2, and ZN3. The fabrication process of the ZnO@NiO heterostructures is presented in Fig. 1. The same process as that used for the deposition of a NiO layer on carbon cloth substrate was used.

### Material Characterization

The morphologies and structures of the ZnO nanorods, NiO nanosheets, and ZnO@NiO heterostructures were characterized by field emission scanning electron microscopy (FESEM; NoVaTM Nano SEM 250, FEI), X-ray diffraction (XRD; Bruker D8 Advance), and transmission electron microscopy (TEM; Tecnai G2 F20, FEI). The surface chemical composition and states of ZN2 were determined using an X-ray photoelectron spectrometer (Thermo ESCALAB 250XI) equipped with a monochromatic Al Kα source (1486.6 eV). Photoluminescence (PL) measurements were carried out with a JY-630 micro-Raman spectrometer using the 325-nm line of a He–Cd laser as the excitation source.

### Photocatalytic Activity

The photocatalytic activity of the samples (ZnO, NiO, and ZnO@NiO) was investigated by examining the photodegradation of rhodamine B (RhB) and methyl orange (MO). The photocatalytic set-up (XPA series-7, Nanjing) was equipped with a 500W mercury lamp as the light source. Typically, the photocatalyst sample grown on the carbon fiber cloth substrate (2 cm × 1.5 cm) was placed into a quartz tube filled with 20 mL of RhB or MO (5 mg/L) aqueous solution. The solution was first kept for 60 min in the dark to ensure an adsorption–desorption equilibrium between the photocatalyst and the dye, after which irradiation with UV light was initiated. At given intervals of illumination, the concentration of the dye was determined by measuring the absorbance of the dye solution at 464 nm (for MO) and 554 nm (for RhB) on a UV-Vis spectrophotometer (TU-1900/1901, Beijing). The experiments were performed at room temperature.

### Photocurrent Response Characterization

All electrochemical measurements were performed using a typical three-electrode system. A 0.5M Na_2_SO_4_ aqueous solution (with pH buffered to ~ 7.0) was used as the electrolyte. A 10W UV lamp was used as the light source for the photocurrent test.

## Results and Discussion

**Fig. 2:**
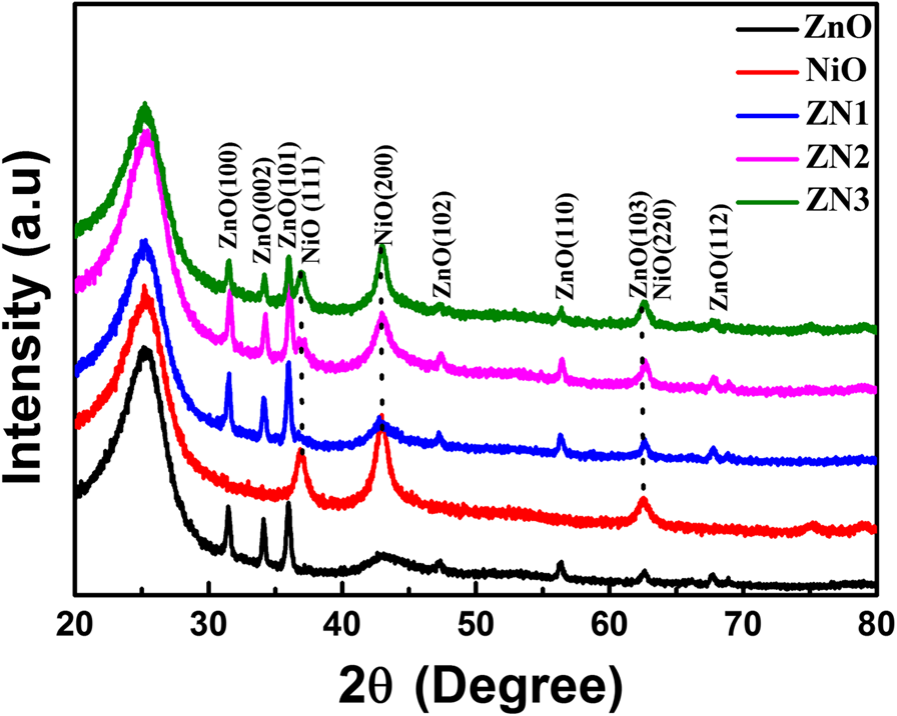
XRD patterns of ZnO, NiO, and ZnO@NiO composites

The XRD patterns of the ZnO nanorods, NiO nanosheets, and ZnO@NiO nanocomposites grown on a carbon fiber cloth are shown in Fig. 2. The broad diffraction peaks located at 25.7° and 43.7° could be ascribed to the carbon cloth. The diffraction peaks observed for the ZnO nanorods could be assigned to the crystal planes (100), (002), (101), (102), (110), (103), and (112) of hexagonal wurtzite ZnO. The diffraction peaks observed at 37.0°and 42.9° in the XRD pattern of NiO nanosheets could be assigned to the crystal planes (111) and (200) of cubic NiO. The XRD patterns of ZN1, ZN2, and ZN3 heterostructures displayed diffraction peaks of hexagonal structure ZnO and cubic structure NiO. Additionally, the diffraction peaks of NiO gradually strengthened as the deposition time for preparing the composite heterostructures increased from 5 to 15 min. Furthermore, no other characteristic peaks were observed, and no crystal phase transformation of ZnO was observed after NiO deposition, confirming the high purity of the composites prepared.

**Fig. 3:**
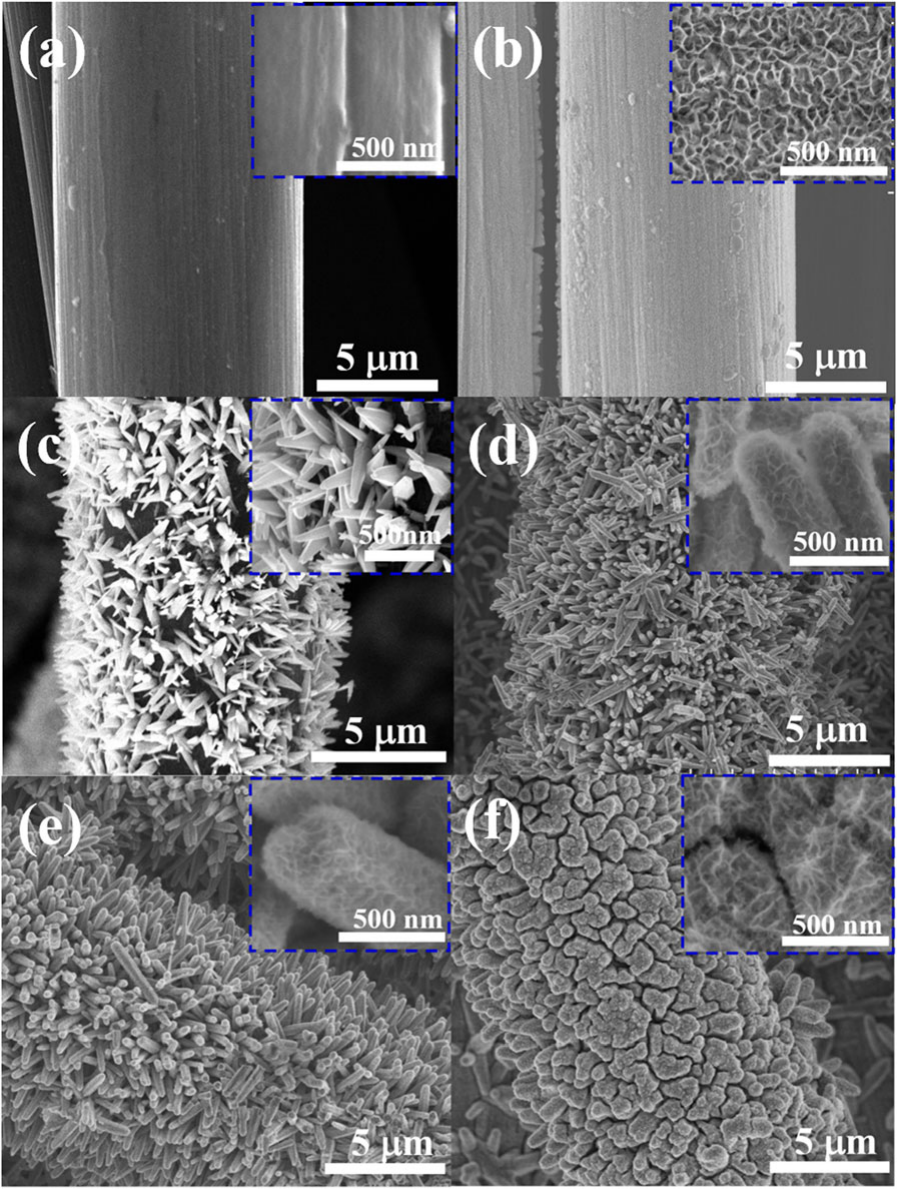
SEM images of **a** carbon cloth, **b** NiO nanosheets, **c** ZnO nanorods, **d** ZN1, **e** ZN2, and **f** ZN3

Figure 3a shows representative top view SEM images of the carbon fiber cloth substrate. The fibers had a smooth surface (inset in Fig. 3a). As observed in Fig. 3b, NiO with a sheet-like structure grew evenly on the carbon fiber cloth. In contrast, ZnO grew as nanorods on the carbon fiber cloth (Fig. 3c). ZnO nanorods with a smooth surface and diameters of 200 nm were obtained in large yield (inset of Fig. 3c). The FESEM images of the ZnO@NiO composites are displayed in Fig. 3d–f. The diameter of the heterostructures became larger upon deposit of NiO nanosheets. Prolonging the deposition time of NiO to 10 min (Fig. 3e) increased the density of the deposited NiO nanosheets. When the deposition time was extended to 15 min (Fig. 3f), the top of nanocomposites was interconnected, indicating further increases in the amount of NiO deposited, as consistent with the XRD results.

**Fig. 4:**
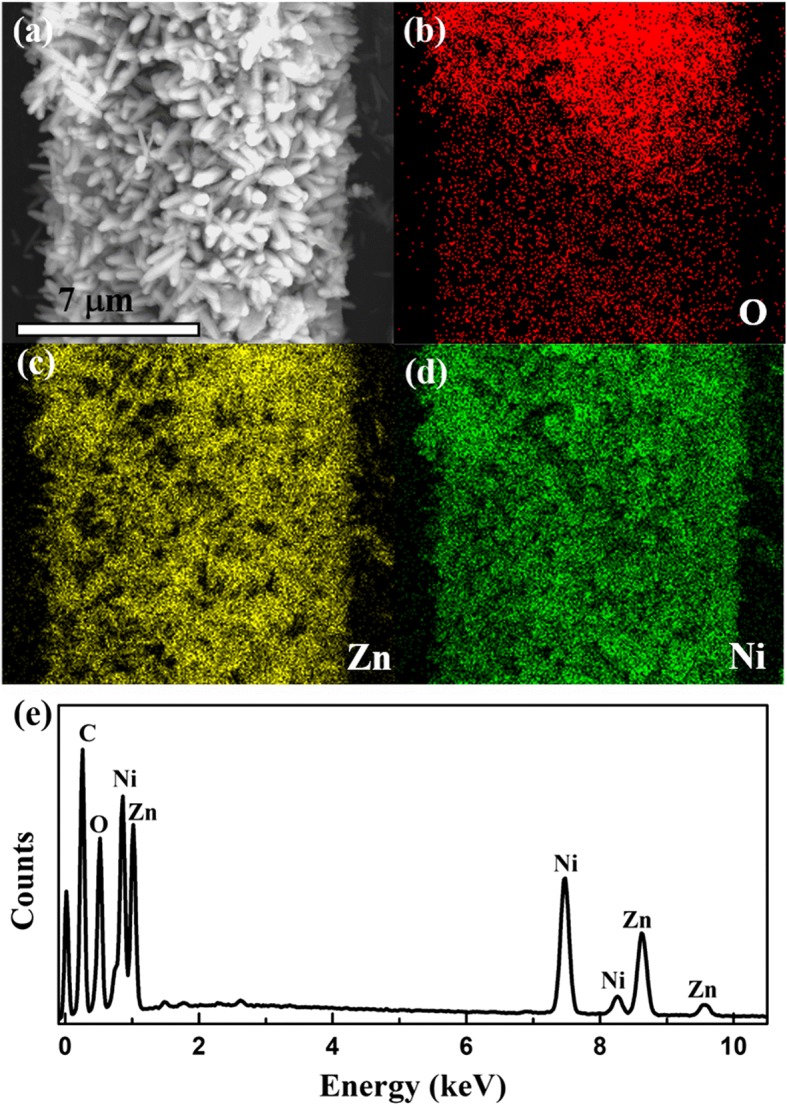
Typical energy dispersive X-ray spectroscopy (EDS) elemental mapping images of ZN2. **a** The corresponding SEM image of the mapping area. **b** O mapping. **c** Zn mapping. **d** Ni mapping. **e** EDS images

The EDS elemental mappings in Fig. 4b and c, corresponding to the SEM in Fig. 4a of ZN2 sample, clearly reveal the uniform spatial distribution of zinc (Zn), nickel (Ni), and oxygen (O) elements, indicating that NiO nanosheets uniformly distributed on the surfaces of ZnO nanorods. The abovementioned elements in hierarchical ZnO@NiO are also confirmed by EDX measurement in Fig. 4, which is consistent with the observations of the SEM.

**Fig. 5:**
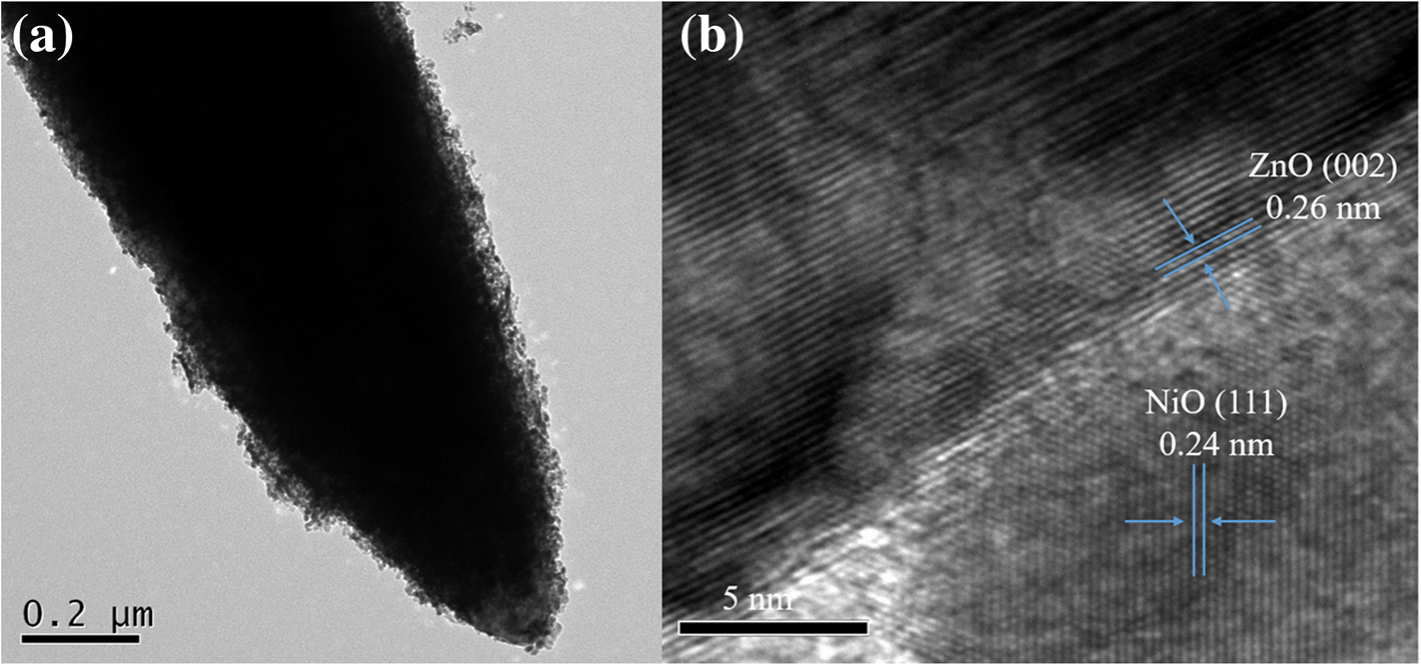
**a** TEM image of ZnO@NiO heterostructure (ZN2). **b** High-resolution TEM of the image of ZN2

As observed in a representative TEM image in Fig. 5, the ZnO@NiO heterostructures (ZN2) have a core–shell structure that consists of ZnO nanorods as the core and NiO nanosheets as the shell. The diameter of the rod-like morphologies was ~ 200–300 nm. The high-resolution TEM image in Fig. 5b shows the interfaces of the crystalline ZnO and NiO crystal lattices. The interplanar distance of 0.26 nm coincides with the lattice spacing of the (002) plane of hexagonal wurtzite ZnO, while the lattice spacing of 0.241 nm corresponds to the interplanar spacing of the (111) plane of cubic NiO. Moreover, the distinct interface and continuity of lattice fringes observed between the NiO and ZnO nanostructures in Fig. 5b indicate the formation of p–n heterojunction between NiO and ZnO in the ZN2 nanostructure.

**Fig. 6:**
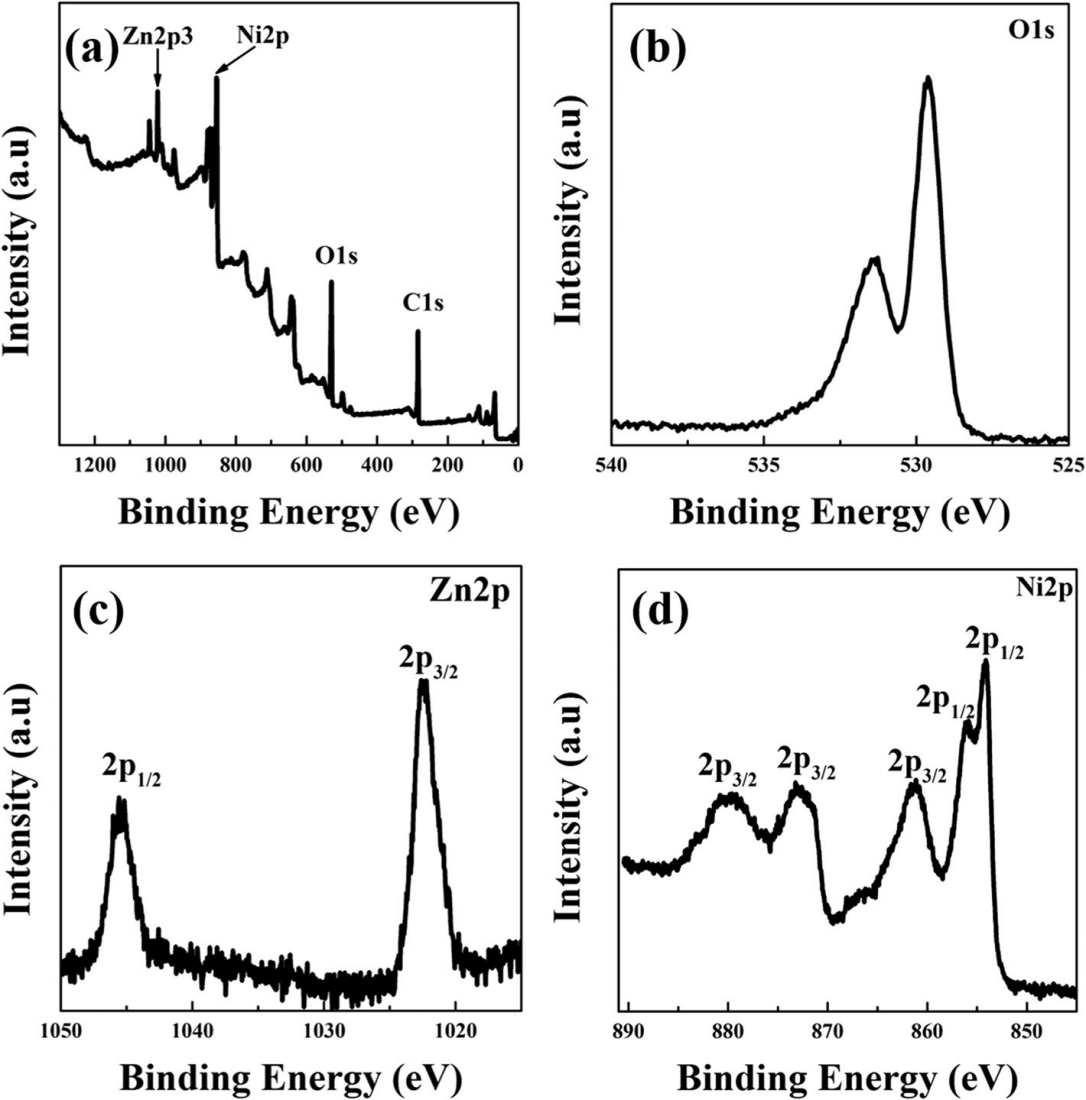
XPS spectra of ZN2. **a** Survey spectra. **b** O 1s. **c** Zn 2p. **d** Ni 2p spectra

X-ray photoelectron spectroscopy (XPS) patterns of ZN2 are shown in Fig. 6. Peaks corresponding to four elements, Zn, O, Ni, and C, were observed in the XPS survey spectra (Fig. 6a). The C1s peak with a binding energy of 284.6 eV was used as the standard reference for calibration and is mainly attributed to hydrocarbon contaminants, typically present in XPS spectra [15]. In Fig. 6b, the XPS peaks located at 529.5 eV were ascribed to the lattice oxygen, whereas the energy peak at 532.2 eV was assigned to non-adsorbed O_2_ or surface hydroxyl species [35]. In Fig. 6c, the two peaks centered at binding energies of 1022.3 and 1045.2 eV were attributed to the Zn 2p_3/2_ and Zn 2p_1/2_ states [36], suggesting that Zn existed in the form of Zn^2+^. Figure 6d shows the Ni 2p XPS signals of ZN2, which could be deconvoluted into five peaks. The peaks at 854.0, 856.1, and 861.1 eV, corresponding to the Ni 2p_3/2_ state, could be ascribed to Ni–O. The remaining two peaks at 873.1 and 879.6 eV were attributed to the Ni 2p_1/2_ state [32].

**Fig. 7:**
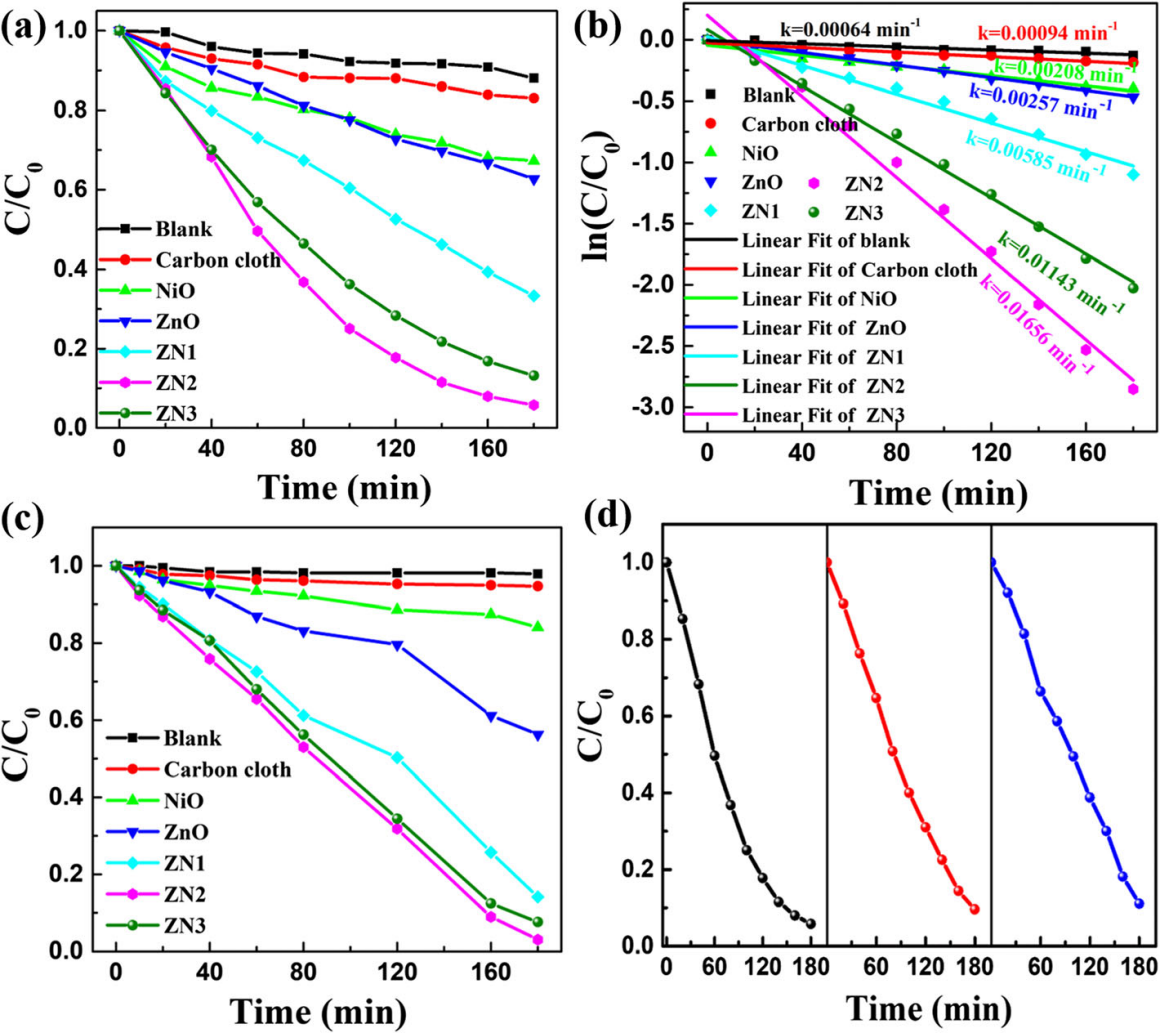
**a** Plots of relative concentration (C/C_0_) of RhB versus time for the degradation of RhB over ZN1, ZN2, ZN3, and ZnO nanorods and NiO nanosheets under UV light irradiation. **b** Corresponding plots of − ln(*C*_*t*_/*C*_0_) versus irradiation time. **c** Plots of relative concentration (*C*/*C*_0_) of MO versus time for the degradation of MO over ZnO, NiO, and ZnO@NiO heterostructure under UV irradiation. **d** Repeated photocatalytic degradation of RhB over ZN2

To investigate the potential applicability of the ZnO@NiO nanocomposites, the photocatalytic activities of the samples were examined by measuring the degradation of RhB dye under ultraviolet light irradiation. The characteristic absorption of RhB at 554 nm was used to monitor its concentration during the degradation process. After 180 min, 95% of RhB was degraded in the presence of ZN2. In comparison, the ZnO nanorods and NiO nanosheets respectively degraded 38% and 33% of RhB only (Fig. 7a). Moreover, the photodegradation activity of the ZnO@NiO nanocomposites was much higher than that of the ZnO nanorods and NiO nanosheets. To measure the photodegradation activity, a plot of the photodegradation rate constant of RhB versus degradation time was used. The reaction can be described as a pseudo-first-order kinetics model as follows [9]:

$$ \ln \left(\frac{C}{C_0}\right)=- kt, $$

where *C*_0_ represents the initial concentration of RhB, *C* refers to the concentration of RhB at different irradiation times *t*, and *k* is the reaction rate constant. The linear plots of ln(*C*/*C*_0_) versus time of the photodegradation of RhB over ZnO, NiO, ZN1, ZN2, and ZN3 are shown in Fig. 7b. The rate constant (*k*) corresponds to the slope of the linear fits. The calculated *k* for the degradation of RhB over ZN2 was 0.01656 min^−1^, which was higher than those calculated for the reactions over ZnO nanorods (0.00257 min^−1^) and NiO nanosheets (0.00208 min^−1^). Overall, the photocatalytic activity decreased in the order of ZN2 > ZN3 > ZN1 > ZnO nanorods > NiO nanosheets. The experimental results suggest that the deposition of the NiO layer on the ZnO nanorods facilitates charge transfer, thus significantly improving the photocatalytic activity. According to the BET results (shown in Additional file 1), the specific surface areas of ZnO@NiO composites initially increase as the increment of deposition time of NiO and then decrease as the deposition time further increases; thus, the ZN2 exhibits the highest photocatalytic activity.

The photocatalytic degradation of MO dye under ultraviolet light irradiation was also examined, and the results are displayed in Fig. 7c. Likewise, the photocatalytic activity decreased in the order of ZN2 > ZN3 > ZN1 > ZnO nanorods > NiO nanosheets. It could be concluded that the ZnO@NiO nanocomposites exhibit superior photocatalytic activity over the ZnO nanorods and NiO nanosheets. The photocatalytic stability of ZN2 was assessed by conducting repeated photocatalytic degradation of RhB under ultraviolet light illumination. As observed in Fig. 7d, the degradation yield remained high (~ 95%) across the repeated cycles, with a slight decrease to 90% observed after the third cycle. These results demonstrate the high photocatalytic efficiency and the reusability of the ZnO@NiO heterostructures, which are important attributes for their practical use in real-life applications in eliminating organic pollutants from wastewater.

**Fig. 8:**
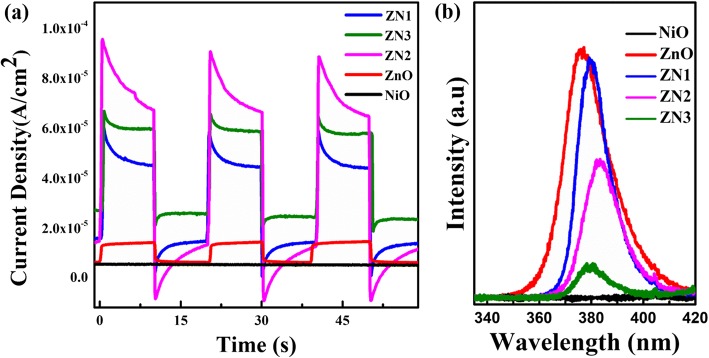
**a** Photocurrent response of NiO nanosheets, ZnO nanorods, and ZnO@NiO heterostructure under UV lamp irradiation (10 W). **b** PL spectra of pure ZnO nanorods, NiO nanosheets, and ZnO@NiO composite

The corresponding photocurrent responses are shown in Fig. 8a. Several on–off light cycles were used to study the separation efficiency of the charge carriers. The NiO nanosheets displayed no changes in the current under both dark conditions and light illumination, whereas the ZnO nanorods showed a small photocurrent response under ultraviolet irradiation. In contrast, the ZnO@NiO composites showed a higher photocurrent density. And the photocurrent density decreased in the order of ZN2 > ZN3 > ZN1 > ZnO nanorods > NiO nanosheets. The fast photocurrent responses implied that charge transport in the samples was very quick. The enhanced photocurrent response of the ZnO@NiO composites can be ascribed to the formation of intimate interfacial contacts between the ZnO nanorods and NiO nanosheets. It is deduced that the photoexcited electrons in the NiO nanosheets can be generated and efficiently transferred from the CB of NiO to the neighboring ZnO nanorods under ultraviolet irradiation, where ZnO serves as an efficient electron sink and transporter, thus inhibiting recombination of photogenerated electron−hole pairs. It is worthwhile to note that the photocurrent of the ZnO@NiO composites initially increased as the deposition time of NiO increased and then decreased as the deposition time was increased further. It is possible that the interfacial contact surface area between the ZnO nanorods and NiO nanosheets initially increases and then decreases as the deposition time of NiO increases, which is consistent with the result of the photocatalytic activity.

Figure 8b displays the typical PL spectra of pure ZnO nanorods, NiO nanosheets and ZnO@NiO heterostructures measured under the same condition at room temperature. For pure ZnO nanorods, a strong emission peak at 378 nm is observed, which is corresponding to the near band edge emission of ZnO. For NiO nanosheets, no emission peak is observed. Furthermore, the PL emission intensity of ZnO@NiO composite is obviously weakened compared with that of pure ZnO nanorods, indicating that the recombination of photogenerated electron–hole pairs is restrained. The results of photocurrent and PL indicate that the ZnO@NiO nanocomposite can remarkably enhance the separation efficiency and interfacial charge transfer efficiency of photogenerated electron–hole pairs.

**Fig. 9:**
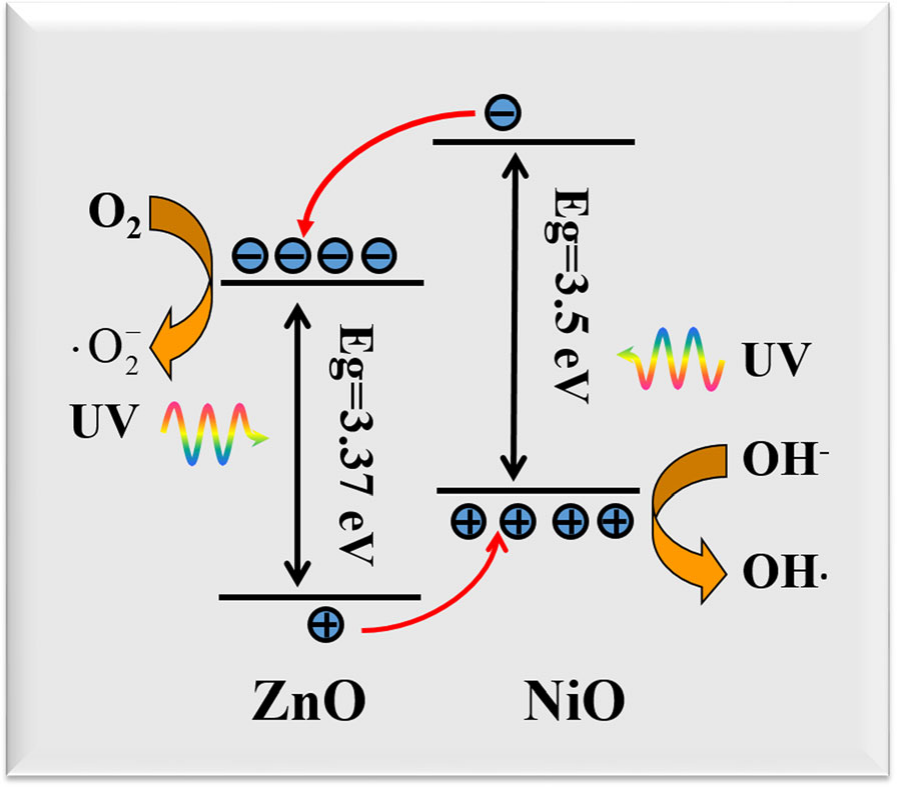
Scheme of the energy band alignment between ZnO and NiO

The improved photocatalytic activity of the ZnO@NiO heterostructures was ascribed to the fast carrier separation and transport at the interface of the ZnO@NiO heterostructures owing to the type II band alignment between ZnO and NiO. This proposed mechanism is consistent with that in previous reports [8, 10, 22]. Figure 9 shows a proposed energy band structure diagram of the ZnO@NiO heterostructure. ZnO is an n-type semiconductor, whereas NiO is a p-type semiconductor. A p–n heterojunction is formed when ZnO and NiO combine, and an inner electric field is generated at the interface between NiO and ZnO because of electron and hole transfers. Under UV light irradiation, the electrons in the VB are excited to the CB, leaving holes in the VB. Band alignment of the p-type NiO and n-type ZnO heterojunctions is beneficial for transferring the photogenerated electrons from the CB of NiO to the CB of ZnO, then the electrons can combine with the dissolved oxygen molecules and produce the superoxide radical anions (•O_2_^−^), which play an important role in the overall photocatalytic reaction. Conversely, the photogenerated holes can transfer from the VB of ZnO to the VB of NiO, and the holes are easily trapped by OH^−^ at the catalyst surface to further yield the hydroxyl radical species (•OH), which is an extremely strong oxidant for decomposing the organic dye. Therefore, the ZnO@NiO nanocomposites exhibited superior photocatalytic performance over the ZnO nanorods and NiO nanosheets.

## Conclusions

ZnO@NiO heterostructures were successfully fabricated by a simple electrochemical deposition method. The photocatalytic activity of the ZnO@NiO nanocomposites was superior to that of ZnO nanorods and NiO nanosheets toward the degradation of MO and RhB dyes under UV light irradiation. The high photocatalytic performance was ascribed to the high separation efficiency of the photogenerated electron–hole pairs from the p–n heterojunction, as confirmed by the photocurrent response measurements. The results showed that more free carriers could be generated and separated in the ZnO@NiO heterostructures, thus leading to higher separation efficiency when compared with that achieved in the ZnO nanorods and NiO nanosheets. Moreover, the ZnO@NiO heterostructures could be easily recycled with minimal decreases of the photocatalytic activity. The high photocatalytic efficiency and reusability of the ZnO@NiO heterostructures, which allows easy separation from the solution, have important applications in eliminating organic pollutants from wastewater.

## Additional file

Additional file 1: **Table S1.** The BET specific surface areas and pores distributions of samples. (DOCX 28 kb)

## Abbreviations

CB: Conduction band
EDS: Energy dispersive X-ray spectroscopy
MO: Methyl orange
PL: Photoluminescence
RhB: Rhodamine B
SEM: Scanning electron microscopy
TEM: Transmission electron microscopy
VB: Valence band
XPS: X-ray photoelectron spectroscopy
XRD: X-ray diffraction
