# Supplementary material for: Fabrication of Hierarchical ZnO@NiO Core–Shell Heterostructures for Improved Photocatalytic Performance
Source: Nanoscale Res Lett. 2018 Aug 30;13:260. doi: 10.1186/s11671-018-2676-1 (PMC6117222; doi:10.1186/s11671-018-2676-1)
Supplement: Supplementary file 1 — Table S1. The BET specific surface areas and pores distributions of samples. (DOCX 28 kb) [file 11671_2018_2676_MOESM1_ESM.docx]

**Fabrication of hierarchical ZnO@NiO core–shell heterostructures for improved photocatalytic performance**

Meng Ding, Hongcen Yang, Tian Yan, Chenggang Wang, Xiaolong Deng, Shouwei Zhang, Jinzhao Huang, Minghui Shao, Xijin Xu*

School of Physics and Technology, University of Jinan, 336 Nanxinzhuang West Road, Jinan, 250022, People’s Republic of China

Table 1s. The BET speciﬁc surface areas and pores distributions of samples.

|  | ZnO | ZN1 | ZN2 | ZN3 |
| --- | --- | --- | --- | --- |
| BET speciﬁc surface area (m^2^/g) | 19.38 | 21.92 | 25.43 | 15.12 |
| Pore radius (nm) | 1.75 | 1.57 | 1.56 | 1.56 |
